# Practical Inquiry on Medicinal Naphtha in Tubercular Phthisis

**Published:** 1845-01

**Authors:** 


### A PRACTICAL ENQUIRY INTO THE VALUE OF MEDICINAL NAPHTHA IN TUBERCULAR PHTHISIS. By *Ed. Oct. Hocken*, M.D. Physician to the Blenheim-street, Infirmary, &c. &c. &c. 8vo. pp. 72. Highley, London, Nov. 1844.

WHENEVER we hear of a remedy for tubercles in the lungs, we feel a scepticism rising in our minds. When recovery takes place, what proof have we that tubercles existed—or that they were removed by any specific medicine? Scarcely a day passes that we do not see and hear young doctors diagnosticate the existence of tubercles in the lungs—often, according to their own account, when they are not larger than pins' heads! In such cases, it is not unlikely that naphtha, or pure air, or Prussic acid—or TIME will effect a cure of "tubercular phthisis." In the early stages of pulmonary tubercles we believe that they cannot be detected, and therefore the vaunted cures are more than doubtful—in the confirmed and obvious cases, the result is not at all doubtful—being DEATH in 99 instances out of 100!

Dr. Hocken truly informs us that auscultation and percussion require "a *natural* delicacy in hearing and appreciating sounds," as well as a "*taught and thoroughly practised ear*" to interpret those sounds. In the succeeding page, he tells us that "he believes his sense of hearing to be naturally *excellent*, as he has found himself capable of distinguishing and appreciating sounds which were not detected by some others engaged in the same pursuits." In addition to all this he informs us that, "during the last ten years, he has possessed very ample opportunities of gaining proficiency as an auscultator"—and that, during this time, "he has studied under some of the best auscultators in the world." We would hint to the ingenious author that, it would have exhibited more "*natural delicacy*," on his part, if he had employed some friend, or one of his master auscultators, to pen the foregoing particulars.

Dr. Hocken commences his preface with the following declaration:—"The author has put forth the following essay, with a sincere desire to benefit *others*, and entirely without the probability of benefitting *self*." This has been the declaration of almost every author who has ushered an infallible remedy for an incurable disease into the world. For what purpose does Dr. Ramadge render "*consumption curable*," but for the benefit of others?

Our author was stimulated to the investigation in question by "the fact that every case of the disease, sufficiently advanced to admit of a positive diagnosis, had invariably progressed from bad to worse, and from this to death." He candidly acknowledges that he commenced the exhibition of naptha with complete disbelief of its medicinal utility in the disease under consideration—" believing this to be, in its nature, incurable." The number of cures which he has accomplished by naphtha raises some sceptical feelings in our breasts, as to the great perfection which Dr. H. has attained in auscultation. The " post hoc ergo propter hoc " argument has done more injury in medicine than any twenty other causes put together. It is known that neither tubercular depositions, nor even tubercular excavations are invariably fatal. But a remedy, in such cases, gets the credit, where Nature was the doctor!

" Several remarkable cases of phthisis,' says Dr. Graves, ' have occurred in my own practice, and in the practice of Dr. Stokes, in which the patients recovered either temporarily or permanently, in a manner quite unforeseen and unexpected. In some recovery took place after the occurrence of abundant tubercular deposition and crepitus, and in others after the formation of tubercular cavities. Facts such as these ought to prevent the practitioner from placing too great reliance upon stethoscopic examinations as a positive means of *prognosis;* for it may be looked upon as established that phthisis, like most other diseases, *does not always necessarily progress to a fatal termination.*" 3.

*Medicinal and Non-Medicinal Naphtha.*—The difficulty of getting real medicinal naphtha is so great, that we almost despair of obtaining this invaluable specific.

" My own observations would lead me to the conclusion that *two-thirds* of those who have tried naphtha in consumption, including *all* those who have found the substance they have employed either useless or injurious, (and have thus apparently failed), *have not* used the medicinal naptha which I have exhibited with constant relief, and in many cases with a curative effect, to my patients—being the same which Dr. Hastings recommends and employs." 5.

The best naphtha that our author has obtained has been at White's (Piccadilly) and Bell's in Oxford-street.*

" The physical characters of the medicinal naphtha are as follows:—When pure it is a *colourless,* limpid liquid, readily miscible in all proportions with water and alcohol, having a specific gravity at 60° F. of about ·800.

" The smell is alcoholic and peculiar—being a compound smell of alcohol and acetic æther, with something rather nauseous and bitter, but *by no means very unpleasant or disgusting.*

" The taste is warm, alcoholic, and rather nauseous—far, however, from being either very unpleasant or disgusting. Patients have invariably told me, that although they could not consider the naphtha pleasant, still it was not at all worse to take than anything else bearing the name of medicine.

" Hence if a liquid be offered for sale of a dark colour, oily appearance, or which becomes milky on the addition of water, it is obviously a non-medicinal specimen; so also if it possess a very nauseous, sickly smell or taste.

" Specimens, however, may be found which agree in all these particulars, and yet belong to the non-medicinal class. I have several times tried a specimen which is more purely alcoholic in its taste and smell, and without the slight nauseous flavour and smell of the medicinal, which invariably disagreed with

* Mr. Bullock, in Conduit-street manufactures the article.—*Rev.*

the patients, aggravated all the symptoms, and produced headache and sickness. These very patients had been previously much benefitted by the medicinal naphtha, and were again speedily relieved from all the distressing symptoms by returning to it, and by leaving off the liquid which disagreed.

"The medicinal naphtha occasions a sense of warmth on being swallowed, usually followed by expulsion of flatus, soon succeeded by a comfortable sense of freedom of respiration, with relief of the distressing shortness of breath so constantly experienced by the phthisical. The peculiar odour of the medicine is imparted to the breath and sputa, and sometimes to the urine passed shortly after its use. In no case have I found the medicinal naphtha derange the stomach or bowels, or produce any unpleasant symptom. Hence if nausea, vomiting or purging, faintness, giddiness or headache follow the exhibition of any liquid used as naphtha, it should be rejected as a non-medicinal article." 6.

After a long disquisition on the chemical characters of the medicinal and non-medicinal naphtha, Dr. H. concludes that "the most ready test is, therefore, to be found in litmus paper, which is reddened by the non-medicinal, but is unaffected by the medicinal." As for the naphtha dissolving tubercles, according to some of its patrons, Dr. H. does not vouch for the fact, because "among the patients who had been taking naphtha, none had died except where the changes of phthisis had been very extensive and very far advanced." This passage alone would excite strong suspicion that those who recovered were not phthisical at all. The following is Dr. Hocken's delineation of the *physical signs* in those patients to whom he has administered the naphtha.

"The *physical signs* in these cases will be found to have chiefly been more or less *flattening* of the upper and anterior part of the chest, from a diminution of the antero-posterior diameter, affecting most commonly the right side alone, then both sides, and less commonly the left side alone; *diminished respiratory play* of the affected portion of the chest; more or less *dulness* and *resistance* on percussion of the sternal end of the clavicle, and of the sub-clavicular region (especially at a little distance from the sternum) of the affected side; increased *resonance* of the *voice* and *heart sounds* over the dull portions; alteration of the normal characters and rhythm of respiration, viz.—*loudness, harshness,* and sometimes *dryness* of both the *inspiratory* and *expiratory murmurs* with *prolongation* of the *expiratory*—sometimes *weakness or suppression of the respiratory murmurs*—these states passing into the slight diffused *bronchophony* and *bronchial breathing,* which increases with the increase of the condensation, till the bronchial characters are more intense, and the voice is transmitted with the characters to which Laennec applied the name of imperfect pectoriloquy. These patients suffer from hectic fever in different degrees, pain in one or both shoulders, cough, generally hæmoptysis, and difficulty or rather shortness of breathing (breathlessness) with mucus expectoration. They have generally near relatives, who are suffering or who have died of consumption, (especially if they have attended much on their sick friends), and are often the children of phthisical parents." 12.

We candidly admit that, if those patients who presented the foregoing phenomena recovered by the use of naphtha, it is the most valuable medicine that has been discovered since the days of Hippocrates. But, the "*delicacy and accuracy*" of our author's ear, and his ability to hear and appreciate sounds which others could not, rise on the mental horizon, and darken the visions of hope that were crowding on our imaginations.

As we cannot take account of the twenty-three cases that are recorded in this brochure, we will quote the first case on the list, as a fair specimen of the rest—some of which, however, were more grave—others less.

" CASE 1. *Tubercular deposition in the upper and anterior portion of the superior lobe of the right lung. Complete recovery from the influence of Naphtha.*—Henry Bonnar, ætat. 23, admitted under my care, at the Blenheim-street Infirmary, on the 2nd of March, 1844, resides at 50, Salisbury-street, Lissongrove. Complains of indisposition, hectic symptoms, pain behind the centre of the sternum, and in the right shoulder, with slight cough, especially in the early morning, with expectoration of transparent mucus. These symptoms have come on gradually during the last two or three months. His father is at present (March) under my care, in an advanced stage of phthisis.

*Physical signs.*—Slight dulness of the sternal end of the clavicle, and of the upper and sternal portion of the sub-clavicular region of the right side on percussion; morbidly increased resonance of the voice and heart-sounds; murmurs harsh, expiration prolonged.

" Ordered Naphthæ Medic. ♏ v ex cyatho aquæ ter die.

" March 9th. Slightly improved. To continue as before.

" March 30th. Chest symptoms relieved; general health much improved. To continue.

" April 6th. Complains of much general indisposition, and pain in the right shoulder. The dulness on percussion is sensibly diminished since the last examination; murmurs less harsh; voice highly resonant.

" To take an ounce three times a-day of the following mixture:—℞. Inf. Gent. Comp. ℥viij, Naph. Med. ʒiij. M., to apply a blister over the sternum.

" April 13th. General and local symptoms improved. To continue, and to produce and keep up an eruption over the front of the chest with the Ung. Autim. Pot. Tart.

" April 20th. Improvement continues.

" May 4th. Has neither pain not uneasiness about the chest or shoulders; cough and shortness of breath greatly relieved; little or no expectoration; still continues to be fatigued, and to perspire freely from slight causes, and is subject to attacks of faintness. The dulness has entirely disappeared; the murmurs are soft and normal, but the voice is still slightly more resonant over the right sub-clavicular region than over the left.

" May 11. Feels perfectly well, and has continued in the enjoyment of perfect health up to the present time (Sept. 1844.)" 13.

In his general conclusions on the cases detailed, Dr. Hocken observes that it is in the chronic forms of the disease that naphtha is most serviceable, " especially where the predisposition to phthisis is not very powerful, and where the tubercular deposition is limited in extent." The earlier the stage of the disease, the greater will be the chance of benefit from the remedy. " Extensive and advanced disease, previous to softening of the tubercles and the formation of caverns, are, *as a general rule,* curable by naphtha." This is by far too sanguine a sentiment. Dr. H. believes that, when excavations have occurred, the disease is incurable.

" The beneficial action of naphtha is seen in the relief of the breathlessness of the patient, the cough, and expectoration; also in the relief of the hectic, the profuse sweating, diarrhœa, &c. Its action on the physical signs is first manifested in the diminution of the dulness on percussion, diminution of the increased resonance of the heart-sounds and voice; a softening down of bronchial breathing into simple harshness of the respiratory murmurs, with a prolongation of the expiratory; then a gradual diminution of these morbid states, and a steady return to the normal murmurs of health." 68.

We would not wish to discourage the trial of any remedy which offers the slightest chance of arresting a disease so destructive to the human race as phthisis is; but from what we have seen of naphtha, and from what we know of the malady, we confess that our expectations are much less ardent than are those of Dr. Hocken, whom we believe to be a young but a talented physician.

